# Identification of GEI-14 as a candidate pro-crossover factor by TurboID in *C. elegans*

**DOI:** 10.17912/micropub.biology.002380

**Published:** 2026-10-01

**Authors:** Yuxin Xi, Yifei Zhao, Ye Hong, Hongtao Zhang

**Affiliations:** 1 Shandong Provincial Key Laboratory of Development and Regeneration, School of Life Sciences, Shandong University, Qingdao, Shandong, China

## Abstract

Previously, our lab used TurboID-based proximity labeling followed by mass spectrometry (MS) to identify proteins that interact with COSA-1 , a cyclin-like pro-crossover factor that localizes at meiotic crossover sites. Here, we investigated potential interactions of COSA-1 with the top 10 COSA-1 ::TurboID-MS candidates ranked by relative intensity. Beyond the established COSA-1 – CDK-2 interaction, AlphaFold 3 predicted strong binding solely between COSA-1 and GEI-14 . Yeast two-hybrid assays confirmed this interaction and further revealed binding of GEI-14 to MSH-5 , another pro-crossover factor. These results demonstrate TurboID's utility in studying the pro-crossover interactome and highlight GEI-14 as a promising candidate for further characterization.

**Figure 1. Identification of GEI-14 as a candidate pro-crossover protein by AlphaFold 3 and yeast two-hybrid assays:** (A) Table listing the top 10 proximity-labeled proteins by COSA-1 ::TurboID-MS. The ranking is based on their relative abundance (percentage of candidate protein intensity relative to the total protein intensity). The data are derived from Liu et al., 2026. For details, please check the supplemental table. All the listed proteins were uniquely identified by COSA-1 ::TurboID (not by the no-TurboID negative control). (B) Bar graph showing the chain-pair ipTM scores for COSA-1 against each of the top 10 candidate proteins listed in (A). (C) Yeast two-hybrid assays showing interactions of GEI-14 with known pro-crossover factors. Positive combinations are highlighted in red. Table: Reagents.

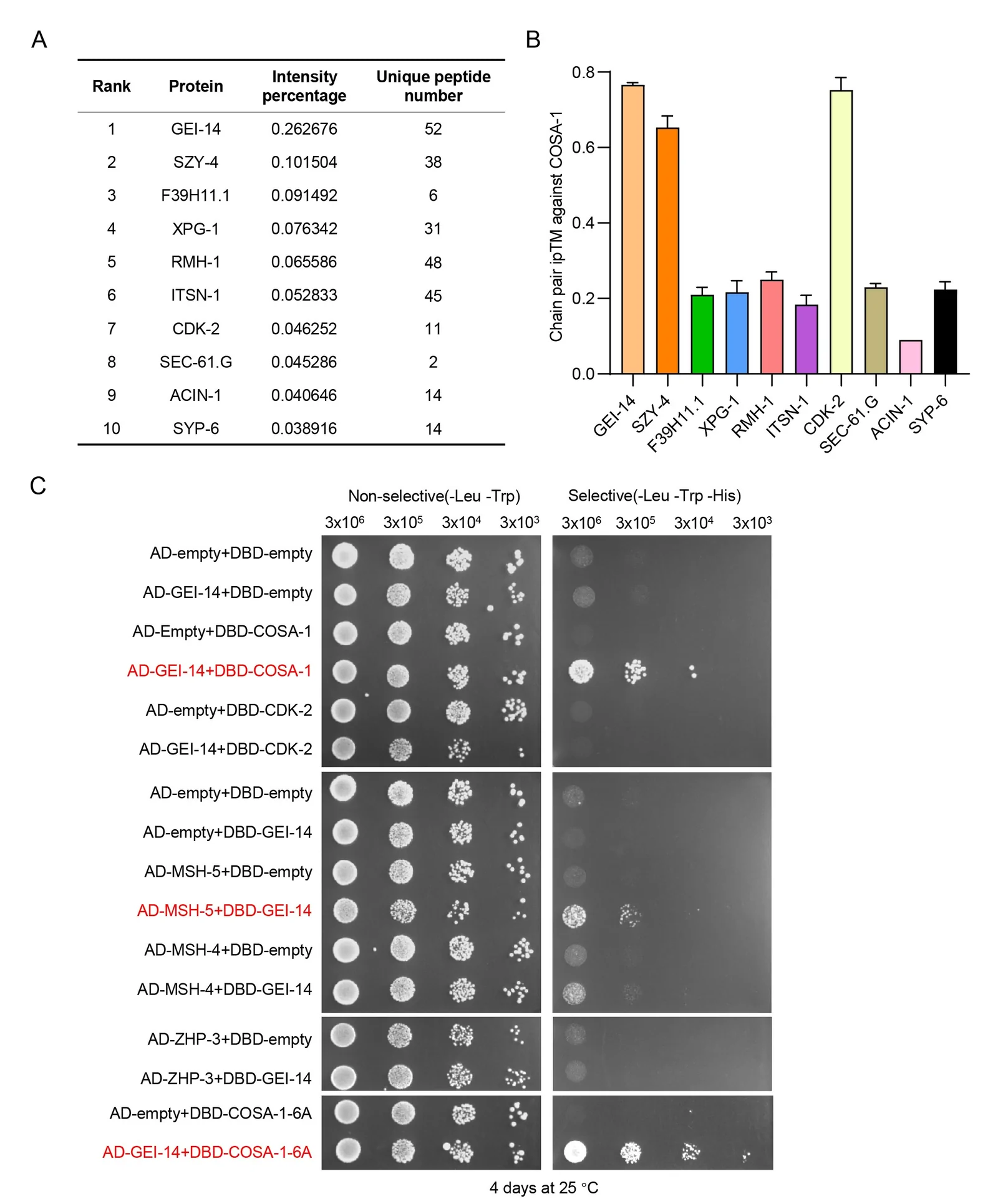



**Table:** 

PLASMID	PROTEIN	VECTOR	AVAILABLE FROM
AD-empty	None	pGADT7	Takara Bio, 630442
DBD-empty	None	pGBKT7	Takara Bio, 630443
AD-ZHP-3	ZHP-3	pGADT7	YH lab, pYH_0094
DBD-COSA-1	COSA-1	pGBKT7	YH lab, pYH_0095
AD-MSH-5	MSH-5	pGADT7	YH lab, pYH_0113
AD-MSH-4	MSH-4	pGADT7	YH lab, pYH_0155
DBD-COSA-1-6A	COSA-1-6A	pGBKT7	YH lab, pYH_0219
DBD-CDK-2	CDK-2	pGBKT7	YH lab, pYH_0358
AD-GEI-14	GEI-14	pGADT7	YH lab, pYH_1278
DBD-GEI-14	GEI-14	pGBKT7	YH lab, pYH_1315

## Description

Accurate segregation of homologous chromosomes into gametes depends on the establishment of interhomolog crossovers during the prophase of meiosis I (Arter and Keeney, 2024). Crossover formation arises from the repair of programmed DNA double-strand breaks (DSBs) via homologous recombination and is directly governed by a set of evolutionarily conserved pro-crossover factors (Gray and Cohen, 2016). These factors can stabilize recombination intermediates and promote their resolution into crossovers (Hollingsworth et al., 1995, Yokoo et al., 2012, Yang et al., 2024, Zhang et al., 2025).

Previously, we employed the TurboID proximity labeling approach to screen for novel interacting proteins of the pro-crossover factor COSA-1 , aiming to gain deeper insight into the mechanism of crossover formation (Yang et al., 2024; Liu et al., 2026). This approach enriched most known pro-crossover proteins, as well as those previously reported to concentrate at crossover-designated sites. It also led us to uncover a role for COSA-1 in bridging crossover designation and Holliday junction resolution. In this follow-up study, we sought to validate potential interactions of COSA-1 with other proximity-labeled proteins identified by COSA-1 ::TurboID-mass spectrometry. The top 10 candidates were selected based on their mass spectrometry-derived spectral intensities after streptavidin affinity purification ( Figure 1A ) (Liu et al., 2026). Among them were proteins with known functions in meiosis, such as CDK-2 , RMH-1 , and SYP-6 . Of note, CDK-2 is a well-established binding partner of COSA-1 (Haversat et al., 2022, Zhang et al., 2025). As a first step, we used AlphaFold 3 to predict potential interactions between COSA-1 and each candidate protein (Abramson et al., 2024), and the resulting modeling revealed that the predicted COSA-1 – GEI-14 complex yielded the highest ipTM score (~0.77) ( Figure 1B ), comparable to that of the COSA-1 – CDK-2 complex (~0.74). This observation prompted us to test this interaction experimentally using yeast two-hybrid assays. Consistent with the prediction, yeast two-hybrid assays confirmed an interaction between GEI-14 and COSA-1 ( Figure 1C ). To determine the specificity of this interaction, other pro-crossover factors were included as controls. No interaction was detected between GEI-14 and CDK-2 , HIM-6 , or ZHP-3 . However, MSH-5 also showed interaction with GEI-14 , albeit weaker than that with COSA-1 ( Figure 1C ). The internal disordered region (IDR) in the N-terminus of COSA-1 has been reported to be essential for its interaction with other pro-crossover factors (Yang et al., 2024). We therefore tested whether the interaction between COSA-1 and GEI-14 is also mediated through this region. Although mutating six amino acids within the IDR of COSA-1(COSA-1-6A) was previously shown to abrogate its binding to MSH-5 or ZHP-3 (Yang et al., 2024), the binding of COSA-1-6A to GEI-14 remained unaffected ( Figure 1C ), implying that the COSA-1 – GEI-14 interaction involves a different region of COSA-1 . Notably, a recent study reported the discovery of GEI-14 as a novel pro-crossover protein through genetic screening (Uebel et al., 2026). That study also found that GEI-14 is required for crossover formation, localizes to crossover sites, and is in close proximity to COSA-1 and accordingly named it COSA-2. Our yeast two-hybrid experiments further validated the physical association between these two proteins and also revealed interactions of COSA-2/GEI-14 with MSH-5. These data reinforce the proposed role of COSA-2/ GEI-14 as a molecular glue that maintains the crossover fate of recombination intermediates (Uebel et al., 2026).

## Methods

For yeast two-hybrid assays, full-length coding sequences for GEI-14 and known pro-crossover factors ( CDK-2 , COSA-1 /COSA-1-6A, MSH-4, MSH-5 , ZHP-3 ) were cloned into plasmid pGADT7 (Takara Bio, 630442) or pGBKT7 (Takara Bio, 630443). Each bait and prey plasmid pair was co-transformed into yeast strain AH109 (Coolaber, CC300). The positive colonies were selected on a medium lacking tryptophan (-Trp) and leucine (-Leu) and were resuspended in 1 ml of 1× PBS. Serial dilutions of the suspension were plated on -His/-Trp/-Leu and -Trp/-Leu solid media, and the plates were incubated at 25°C for 3–4 days before the results were recorded.

## Data Availability

Description: Table listing the top 10 proximity-labeled proteins by COSA-1::TurboID, including two known pro-crossover factors, alongside four additional bona fide pro-crossover factors captured in the same assay and their rank. The ranking is based on their relative abundance. Resource Type: Dataset. DOI: https://doi.org/10.22002/cxkmy-4cv26
